# A Wearable Sensor System to Measure Step-Based Gait Parameters for Parkinson’s Disease Rehabilitation

**DOI:** 10.3390/s20226417

**Published:** 2020-11-10

**Authors:** Niveditha Muthukrishnan, James J. Abbas, Narayanan Krishnamurthi

**Affiliations:** 1 Center for Adaptive Neural Systems, School of Biological and Health Systems Engineering, Arizona State University, Tempe, AZ 85287, USA; Niveditha.Muthukrishnan@asu.edu (N.M.); James.Abbas@asu.edu (J.J.A.); 2 Edson College of Nursing and Health Innovation, Arizona State University, Phoenix, AZ 85004, USA

**Keywords:** spatiotemporal gait, step length, step time, inertial measurement units, gait event detection

## Abstract

Spatiotemporal parameters of gait serve as an important biomarker to monitor gait impairments as well as to develop rehabilitation systems. In this work, we developed a computationally-efficient algorithm (SDI-Step) that uses segmented double integration to calculate step length and step time from wearable inertial measurement units (IMUs) and assessed its ability to reliably and accurately measure spatiotemporal gait parameters. Two data sets that included simultaneous measurements from wearable sensors and from a laboratory-based system were used in the assessment. The first data set utilized IMU sensors and a GAITRite mat in our laboratory to monitor gait in fifteen participants: 9 young adults (YA_1_) (5 females, 4 males, age 23.6 ± 1 years), and 6 people with Parkinson’s disease (PD) (3 females, 3 males, age 72.3 ± 6.6 years). The second data set, which was accessed from a publicly-available repository, utilized IMU sensors and an optoelectronic system to monitor gait in five young adults (YA_2_) (2 females, 3 males, age 30.5 ± 3.5 years). In order to provide a complete representation of validity, we used multiple statistical analyses with overlapping metrics. Gait parameters such as step time and step length were calculated and the agreement between the two measurement systems for each gait parameter was assessed using Passing–Bablok (PB) regression analysis and calculation of the Intra-class Correlation Coefficient (ICC (2,1)) with 95% confidence intervals for a single measure, absolute-agreement, 2-way mixed-effects model. In addition, Bland–Altman (BA) plots were used to visually inspect the measurement agreement. The values of the PB regression slope were close to 1 and intercept close to 0 for both step time and step length measures. The results obtained using ICC (2,1) for step length showed a moderate to excellent agreement for YA (between 0.81 and 0.95) and excellent agreement for PD (between 0.93 and 0.98), while both YA and PD had an excellent agreement in step time ICCs (>0.9). Finally, examining the BA plots showed that the measurement difference was within the limits of agreement (LoA) with a 95% probability. Results from this preliminary study indicate that using the SDI-Step algorithm to process signals from wearable IMUs provides measurements that are in close agreement with widely-used laboratory-based systems and can be considered as a valid tool for measuring spatiotemporal gait parameters.

## 1. Introduction

Spatiotemporal gait parameters have been used to assess gait in healthy populations and those with pathologies [1,2,3,4,5,6,7]. These biomechanical measures can help to characterize gait impairments, serve as a biomarker to monitor the progression of the disease over time [4,8,9], predict falls [10], and to develop targeted gait rehabilitation programs [11,12,13]. Although gait feedback systems based on wearable sensors would provide opportunities to conduct gait training and assessments outside of a lab/clinical setting, only very few such systems exist [14,15]. For calculation of spatiotemporal gait parameters from data derived from wearable inertial measurement units (IMUs), several algorithms have been investigated, such as: wavelet transforms to identify gait events followed by the use of a double pendulum model to calculate stride length [6,7,16]; sensor fusion algorithms that use Magnetic Angular Rate Gravity (MARG)-based adaptive-gain filters, Kalman filters, and extended Kalman filters [17,18] to estimate kinematic parameters such as gait phases, velocity, and position; and artificial neural network-based algorithms to classify the healthy and pathological population and estimate gait features such as minimum toe clearance and gait velocity [19,20,21]. These algorithms [6,7,17,18,19,20] have also been used to calculate gait parameters such as velocity and stride length.

People with Parkinson’s disease (PD) exhibit more variable gait patterns and higher left-right step asymmetry than people without PD [22,23], and these characteristics increase the risk of falls [10,24]. These step-based measures are important biomarkers for indicating the severity of motor impairment and gait asymmetry in people with PD [3,25]. Rehabilitation programs that use real-time feedback of these step-based measures [13] or other gait parameters have been demonstrated to help modulate gait performance and achieve targeted gait characteristics in people with PD [26,27]. Given the gait asymmetries often observed in PD, gait measures that are specific to one side (step-based parameters such as step length or step time), might be more useful for real-time feedback than parameters that are defined by considering movements of both sides, such as stride length or stride time. However, to the best of our knowledge, there are only two previous reports [3,28] of using IMU data to estimate step length of people with PD and comparing the accuracy with a lab-based reference system. These reports demonstrated good agreement in the lab, but reliance on the inverted pendulum model, the need for precise sensor placement, and susceptibility to sensor movement may limit its accuracy when used outside of the laboratory environment.

In this work, we developed a segmented double-integration algorithm to estimate step length and step time (SDI-Step) using data obtained from wearable IMUs. Our SDI-Step algorithm utilizes several techniques derived from the literature for specific calculations and integrates them into an overall system that is suitable for real-time implementation on lightweight body-mounted processors. Using data obtained from wearable sensors worn by subjects with PD and others without neurological conditions, the accuracy of the SDI-Step algorithm was evaluated by comparing estimates of gait parameters to results obtained from validated laboratory-based systems.

## 2. Materials and Methods

### 2.1. Segmented Double Integration-Step Algorithm

In general, IMU-based gait measurement algorithms can be grouped into three categories, i.e., those that use an abstraction model, a gait model, or direct integration [29]. Abstraction model-based algorithms use neural networks or machine learning (ML) methods to estimate the walking patterns without explicitly using biomechanical principles [30]. Algorithms that use gait models directly utilize biomechanical principles with a predefined model, such as the double pendulum model [28,31]. The accuracy of gait model-based methods relies on predefined generic models that do not account for individual variations in the participant population [29,32]. The large size of the data sets required to train ML models and/or the experimental setup required for data collection can be cumbersome.

Algorithms based on direct integration, which use measures of acceleration derived from IMUs to estimate gait parameters, can be computationally efficient and may be more suitable for wearable systems. Some of these direct integration algorithms also utilize information from gyroscopes, magnetometers, and accelerometers in various stages of the processing to perform high-frequency noise reduction, stride segmentation, sensor alignment, and drift compensation [33].

**Figure 1:**
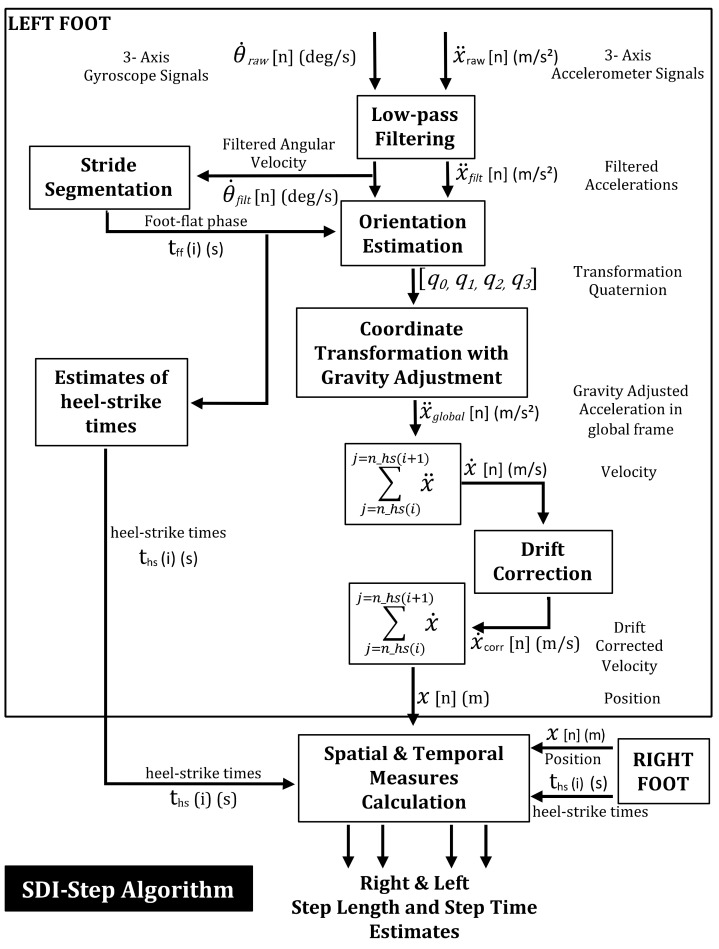
Flowchart depicting the computational modules involved in the Segmented Double Integration (SDI)-Step algorithm developed for an Inertial Measurement Unit (IMU)-based wearable gait measurement system.

In this work, we implemented a direct integration algorithm with additional processing to calculate spatiotemporal gait measures. We used data from two wearable IMU sensors with one placed on each foot of participants. Signals recorded from the accelerometers and gyroscopes of the IMUs were used to estimate step length and step time using the procedures outlined in Figure 1 and described below:

1. **High-frequency noise removal:** The raw data from each IMU sensor was sampled at 128 Hz. The sampled acceleration and angular velocity were passed through a Butterworth low-pass filter with 4 Hz cut-off frequency to remove high-frequency fluctuations from the data [34,35].
2. **Stride segmentation:** The IMU data was then segmented into two phases of gait movements–stationary (foot-flat) phase and non-stationary (swing phase). The time point at which the filtered angular velocity signal crossed a pre-specified threshold (20 degrees/s) was used to detect the onset of swing phase as this threshold was observed to be the lowest value that avoided false positive identification of swing phase initiation. Segmentation was performed to obtain the start and end times of each phase.
3. **Orientation estimation:** A complementary filter [36] was used for orientation estimation, which fuses the static low-frequency linear movements from the accelerometer with the dynamic high-frequency angular movements of the gyroscope.
4. **Coordinate transformation with gravity adjustment:** The acceleration vector of the sensor was transformed to the global coordinate system using the estimated orientation function and the gravitational component of the transformed acceleration was removed.
5. **Heel-strike detection:** The heel-strike events were identified by determining the minima of the angular velocity vector within an average estimate of stride duration for the population.
6. **Velocity estimation:** The velocity of each swing phase was obtained by numerically integrating the gravity-adjusted acceleration between two consecutive heel strikes for each foot.
7. **Drift correction:** Drift in the computed velocity was corrected using a zero velocity update algorithm [37]. The average amount of drift during the foot-flat phase was subtracted from the subsequent swing phase for each step to obtain the drift-corrected velocity.
8. **Position estimation:** The drift-corrected velocity signal was numerically integrated between two consecutive heel strikes to estimate the location (in three dimensions) of each foot at each heel strike.
9. **Step length and step time calculation:** The step length and step times for each foot were obtained using the estimated positions and timings of subsequent heel-strikes.

### 2.2. Participants

The SDI-Step algorithm was evaluated using data obtained from experiments with 20 participants: 14 young able-bodied participants with no neurological deficit (YA) and 6 with a diagnosis of PD. Step lengths and step times obtained by processing data from wearable IMU sensor data with our SDI-Step algorithm were compared with values obtained using a widely-used laboratory-based system, either the GAITRite mat (CIR Systems Inc. Clifton, NJ, USA) or the Elite optoelectronic system (BTS-Bioengineering, Milan, Italy). The PD data set was derived from experiments in our laboratory (Center for Adaptive Neural Systems, Arizona State University) with 6 participants with PD (3 females, 3 males, age 72.3 ± 6.6 years) with disease duration of 5.5 ± 2.1 years and Unified Parkinson’s Disease Rating Scale (UPDRS) score of 40.3 ± 18.2. A portion of the YA dataset was also collected from experiments in our laboratory with 9 healthy young adults (YA_1_) (5 females, 4 males, age 23.6 ± 1 years). In these experiments, participants walked on the GAITRite mat while wearing an IMU (Opal V2, APDM Inc., Portland, OR, USA) on each foot; data were recorded simultaneously from the GAITRite and IMU systems. The second data set, which was drawn from a publicly available site, was collected by Pierleoni et al. [38] with 5 young healthy adults (YA_2_) (2 females, 3 males, age 30.5 ± 3.5 years) using an IMU (X-io Technologies, Bristol, UK) placed on each foot and walking movements simultaneously measured using the Elite optoelectronic system for subsequent calculation of gait measures. For the experiments with the YA_1_ and PD participant groups, all study procedures were approved by the Institutional Review Board at Arizona State University, and all participants voluntarily signed the informed consent form before participating in the screening process. The data utilized as the YA_2_ group were collected by Pierleoni et al. and are publicly available [38].

### 2.3. Measurement Systems and Calculation of Gait Parameters

Wearable wireless IMUs (APDM Opal V2 sensors, APDM Inc., Portland, OR, USA) were used for data collection in the YA_1_ and PD participants. The dimensions of each IMU are 43.7 × 39.7 × 13.7 mm, with weight less than 25 gms. Each IMU consists of a tri-axial accelerometer (17.5 bit/axes), a tri-axial gyroscope (16 bit/axes), and a tri-axial magnetometer (12 bits/axes). The data from each IMU were sampled at 128 Hz, and streamed wirelessly to a PC. The data sets were processed offline and IMU-based gait parameters were calculated using the SDI-Step algorithm described above (Figure 1), which was implemented in MATLAB R2018a (Mathworks, Natick, MA, USA). A GAITRite electronic pressure-sensitive mat (CIR Systems Inc., Clifton, NJ, USA) with the manufacturer-supplied software was used as a lab-based reference system to calculate spatiotemporal gait measures for comparison with IMU-based gait parameters. The mat was connected to a USB port of a computer with GAITRite software, and data were sampled at 100 Hz.

Data from the YA_2_ participants were derived from wearable IMUs (X-IMU, NGIMU, X-io Technologies Limited, Bristol, UK) [38]. The dimensions of each IMU are 57 × 38 × 21 mm, and weigh about 49 g. Each X-IMU sensor includes a tri-axial accelerometer (16 bit/axes), gyroscope (16 bit/axes), and magnetometer (12 bit/axes); data were sampled at 128 Hz and transmitted using a Bluetooth module. For this data set, the lab-based reference system was an optoelectronic system (Elite, BTS-Bioengineering, Milan, Italy) composed of 6 infrared cameras, 8 reflective markers, and an acquisition unit that sampled data at 100 Hz. Our SDI-Step algorithm was applied to the IMU data to calculate gait parameters, which were then compared with those obtained by processing the data from the optoelectronic system. The timing of each heel-strike was identified by determining the time of the vertical minimum of the heel marker after each swing phase and then the corresponding anterior-posterior position of the heel marker was recorded. These values were subsequently used to calculate step time and step length.

### 2.4. Gait Assessment Protocol

The experimental protocol was designed to simultaneously collect data from wearable sensors and a validated lab-based system as subjects walked in a straight line; the accuracy of the SDI-Step algorithm was evaluated by comparing estimates of gait parameters to results obtained from the laboratory-based system. The YA_1_ and PD participants completed six trials on the GAITRite mat while wearing IMU sensors on the dorsum of each foot, over the shoe. The participants were asked to walk at their preferred walking speed with a short break between the trials. The raw data from the IMU’s accelerometer and gyroscope were wirelessly streamed to a laptop and processed offline. To identify the onset of the gait movements using the data collected from the IMUs and GAITRite, each participant completed a few seconds of quiet standing at the beginning of each trial. The computed step length and step time values from the IMU data for each step were compared to the corresponding values obtained from the GAITRite system.

For the YA_2_ participants, an IMU sensor was placed on the dorsum of each foot, and four reflective markers were placed on the following anatomical landmarks of each foot: the heel, the first and fifth metatarsals, and the dorsum of the foot above the IMU sensor. The participants completed a specific path consisting of an 11 m walkway, which started with three heel rises at the beginning of each trial to synchronize data obtained from IMUs with that of the optoelectronic system [38]. All participants performed three repetitions of a specified path within the capture volume with a 20-s break between the trials. Step length and step time parameters were computed using our SDI-Step algorithm from the recorded IMU data and compared to the values calculated from the optoelectronic reference system.

### 2.5. Statistical Analysis

All the data analysis for this validation study was performed using SPSS^®^ (version 24, IBM Corp., Armonk, NY, USA) and MATLAB software (version 2018a, Mathworks Inc., Natick, MA, USA). The agreement analysis (SDI-Step vs. GAITRite for PD & YA_1_; SDI-Step vs. Optoelectronic system for YA_2_) was carried out using the Passing–Bablok (PB) regression analysis, which is a statistical procedure that does not make assumptions about the distribution of data, provides confidence intervals for slope of regression line and intercept, and is insensitive to the distribution of errors and outliers [39,40,41]. Some of the previous literature utilized ICC (2,1) to compare gait parameters from IMU-based measurements and lab-based systems. Thus, to facilitate comparing our results with those of prior reports, we calculated ICC (2,1) to examine the agreement between step lengths and times obtained using SDI-Step and lab-based systems. Moreover, Bland–Altman (BA) plots were used to identify the systematic differences between parameters calculated using SDI-Step and lab-based systems, if any [42].

## 3. Results

**Figure 2:**
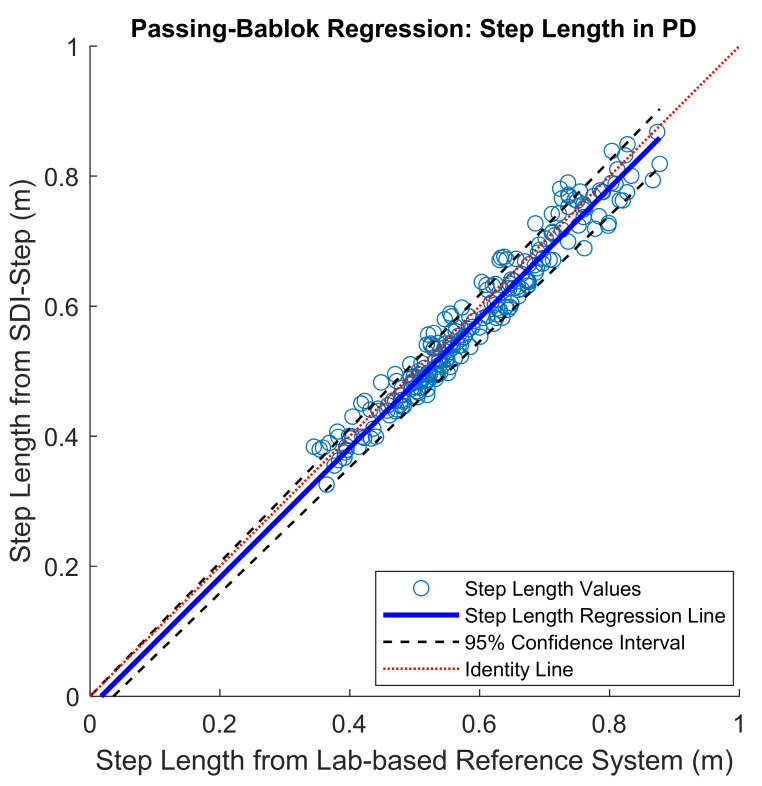
Scatter plots with Passing–Bablok (PB) regression line (blue) along with 95% confidence interval (black dashed line) and identity line (indicated by a red line) for step length values obtained from people with Parkinson’s Disease (PD). PB-slope (95% CI): 1.05 (1–1.17), and PB-Intercept (95% CI): −0.05 (−0.01–0.1).

**Figure 3:**
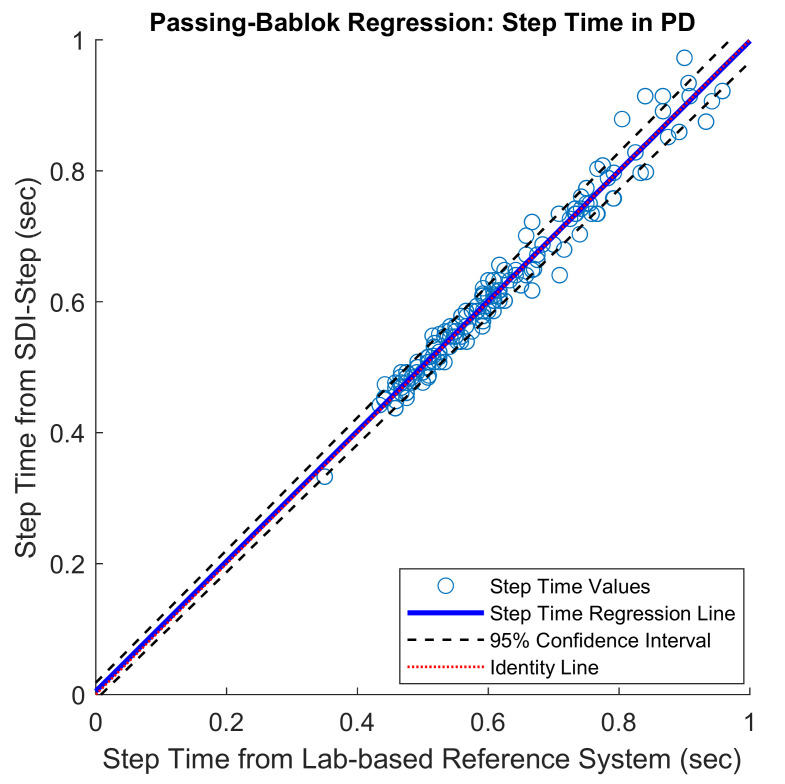
Scatter plots with Passing–Bablok regression line (blue) along with 95% confidence interval (black dashed line) and identity line (indicated by a red line) for step time values obtained from people with PD. PB-slope (95% CI): 1.0 (1–1.16), and PB-Intercept (95% CI): 0 (−0.09–0).

**Figure 4:**
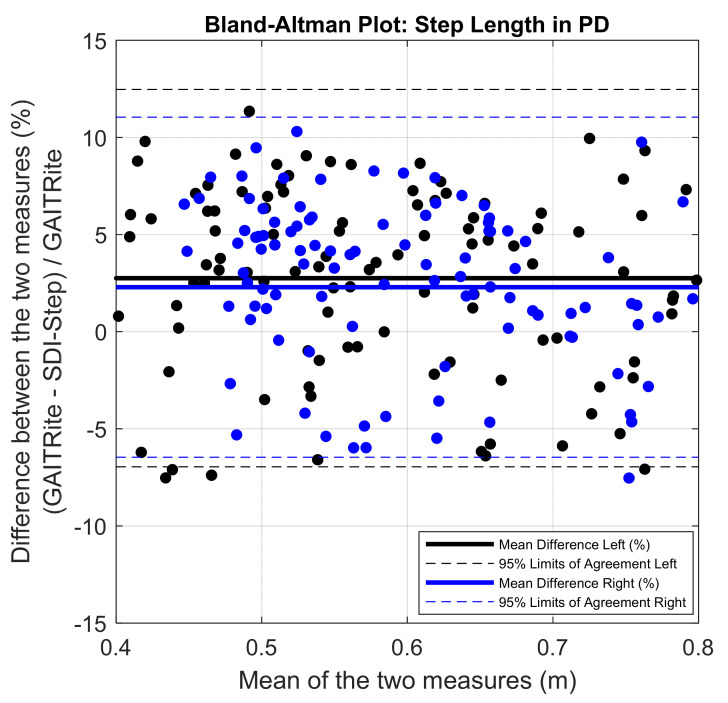
Bland–Altman plots demonstrating agreement between GAITRite and the SDI-Step Algorithm for right (blue) and left (black) step length in PD. Solid line represents the mean difference in step length between the two measures in percentage.

**Figure 5:**
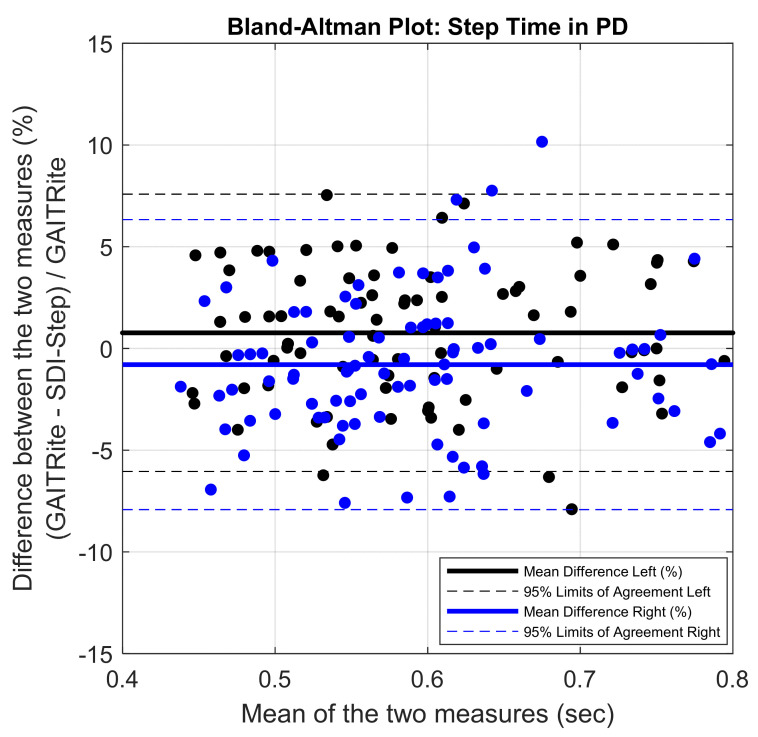
Bland–Altman plots demonstrating agreement between GAITRite and IMU SDI-Step Algorithm for right (blue) and left (black) step time in PD. Solid line represents the mean difference in step time values between the two measures in percentage.

The mean of the errors for step length and step time measures obtained between the IMU system and the lab-based reference system were small (mean error in % for step length = 4.5 ± 2.54; for step time = 2.97 ± 2.51). The PB regression analysis resulted in a slope value close to one and intercept close to zero with the 95% confidence interval including the values one and zero for slope and intercept, respectively, for the comparison of results from the SDI-Step algorithm and reference systems in all the three groups, as shown in Figure 2 and Figure 3 and Table 1. A value of slope equal to one together with a value of an intercept equal to zero indicates a perfect agreement. The ICC (2,1) absolute agreement for step length showed excellent agreement for PD (0.93–0.98), moderate to an excellent agreement for YA_1_ (0.83–0.95), and moderate to an excellent agreement for YA_2_ (0.81 and 0.90), while all the three groups of participants had an excellent agreement for step time ICCs (≥0.9). Finally, visual examination of the agreement using the BA plots showed no systematic error in the measures and the measurement difference was within the limit of agreement (LoA) with a 95% probability (Figure 4 and Figure 5).

## 4. Discussion

This study evaluated the accuracy of step length and step time calculated using the SDI-Step algorithm from IMU-derived measurements by comparing it to the corresponding values obtained from lab-based systems. The findings indicate that the SDI-Step algorithm can produce estimates that are accurate and reliable from wearable sensors. The mean of the errors for step length and step time measures obtained between the IMU system and the lab-based reference system were small, and the algorithm performed well with two different IMU sensor models.

Inspection of the PB regression plots indicates excellent agreement for step time and step length in the PD, YA_1_, and YA_2_ groups, with the values for slope = 1 and intercept = 0 falling within the 95% confidence interval, as reflected by the values reported in Table 1. The ICC (2,1) absolute agreement, a metric that is frequently reported in validation studies, was interpreted according to Fleiss, Levin & Paik criteria [43]. ICC values in the range from 0.83–0.98 suggest that the IMU system is a valid measurement system for both step length and step time parameters in all three groups [25,39].

The results from the Bland–Altman plots shown in Figure 4 and Figure 5 indicate excellent agreement between the gait parameters computed using SDI-Step algorithm and reference systems, and the difference was within the 95% confidence interval. Further inspection of the BA plots indicates that the SDI-Step algorithm underestimated step length and step time parameters in all groups except for step time in the YA_1_ participants. Moreover, the distribution of the errors, as shown in Figure 4 and Figure 5 indicates that there are no systematic differences between the measures calculated using the SDI-Step and lab-based reference system with respect to the magnitude of the mean of the measures.

Some reports of algorithms that calculated gait parameters from IMU data have utilized a simplified inverted pendulum model of gait to estimate parameters [2,3,44], but the gait measures derived from these methods may be adversely affected by errors intrinsic to the specific model formulation. Notably, the inverted pendulum method assumes a straight-line walking at a constant pace with a compass-like gait pattern and these methods also employ a generic correction factor that is not tailored to an individual’s gait characteristics [2,3,44]. Methods based on abstract models typically have high computational demands that may not be feasible for real-time calculation by a wearable processor [36], and often require training on data sets from a large population and/or a comprehensive data set from each user, which may limit widespread use.

We chose to use an implementation of the double integration method with specific modules in the algorithm to improve estimates of step length and step time. We used a complementary filter for orientation estimation to improve estimates of movement during the swing phase. We updated the calibration of zero velocity at every step to correct for sensor integration drift. This reduced the magnitude of error due to drift accumulation and may help with real-time estimation of step length and step time. Most importantly, we used bilateral measures from multiple sensors for gait event detection to delineate segments of the gait cycle for calculation of step time and step length. Our evaluation, which notably included participants with PD and those without neurological conditions, exhibited excellent agreement with lab-based measures in all groups (ICC (2,1) ~ 0.9) for step length and step time, which demonstrates better performance than previous reports with other systems [2,3,45].

Our explicit focus on measurements of step parameters, rather than stride parameters, also influenced the strategy for sensor placement and the selection of specific features in the SDI-Step algorithm. Bilateral placement of sensors is more cumbersome for the user than a single sensor on the torso, but provides much more detailed and reliable information about the movement of each limb. Placement on the foot (rather than on the thigh or lower leg) provides a distinct zero-velocity phase during foot-flat that enables step-by-step drift correction and accurate detection of gait events [46]. Data from each foot are processed separately, and the metrics are aggregated after the integration phase rather than in raw data analysis, similar to the work described in Tunca et al. [46]. This foot-specific tracking simplifies error estimation and correction without influencing the estimates from the other foot.

Previous literature on the validity and reliability of gait parameters calculated from IMU data were predominantly tested on healthy young adults and populations with mild motor-impairment. The results reported here demonstrate that the SDI-Step algorithm can provide valid gait parameters in both the healthy individuals and people with PD who exhibit less prominent heel strikes and significant asymmetry in step length and step time.

The ability to accurately measure step length and step time will enable investigation of rehabilitation strategies that utilize real-time feedback of step parameters [13] to address gait deficits such as reduced step length and spatial and temporal asymmetry due to PD or other conditions. The fact that this SDI-Step algorithm can provide accurate estimates of step parameters from wearable sensors will enable gait monitoring and real-time feedback over long periods in everyday environments such as the home or community.

However, the implications of the present study may be limited by the small sample size used for evaluation and the fact that the data were derived from studies in a laboratory setting in which participants walked on a straight-line path. To more fully characterize the robustness of the system and its potential utility in home and outdoor environments, its performance should be evaluated in a larger and broader cohort and under a variety of conditions, such as different floor surfaces with changes in elevation and different walking patterns with changes in direction, speed, and step characteristics.

## 5. Conclusions

This study in healthy and neurodegenerative populations indicates that the SDI-Step algorithm can utilize data from wearable IMU sensors to provide measurements of spatiotemporal gait parameters that are in excellent agreement with those from widely-used laboratory-based systems. This study in healthy and neurodegenerative populations indicates that the SDI-Step algorithm can utilize data from wearable IMU sensors to provide measurements of spatiotemporal gait parameters that are in excellent agreement with those from widely-used laboratory-based systems. However, the outcomes of the study should be considered preliminary since the study tested the algorithm only in a small sample of people with PD and the robustness of the algorithm should be tested in a larger sample under a variety of gait conditions. Notably, the ability to accurately measure step length and step time for each leg makes this technique particularly suitable for measuring gait asymmetry in real-world environments. These gait measurements from wearable IMU sensors may enable researchers to conduct experiments outside of a laboratory setting and enable clinicians to monitor gait patterns and/or provide gait training therapy that uses real-time feedback in a home environment or in a community setting.

## Figures and Tables

**Table 1:** The step length and step time gait measures calculated using SDI-Step are compared to measurements from the lab-based reference system (GAITRite for YA_1_ and PD participants and Motion capture system for YA_2_ participants). The degree of agreement between our algorithm and the reference systems calculated using various agreement methods are also provided. The BA-bias was collected by subtracting the value calculated using the SDI-Step algorithm from the value obtained from the reference system. The units of mean, SD, PB Intercept with CI, and BA-bias with LoA for step length and step time are in meters and seconds, respectively. SDI-Step: Segmented Double Integration-Step, BA: Bland–Altman plot, ICC: Intra-class Correlation Coefficient, PB: Passing–Bablok regression, CI: Confidence Interval, LoA: Limits of Agreement, SD: Standard Deviation, PD: Parkinson’s disease, YA_1_: Young Adults (Group 1), YA_2_: Young Adults (Group 2).

Descriptive and Statistical Measures	Step Length			Step Time		
	PD (n = 211 steps)	YA_1_ (n = 222 steps)	YA_2_ (n = 160 steps)	PD (n = 211 steps)	YA_1_ (n = 222 steps)	YA_2_ (n = 160 steps)
Mean (SD) Lab-based system	0.59 (0.11)	0.68 (0.09)	0.67 (0.05)	0.73 (0.26)	0.59 (0.06)	0.64 (0.05)
Mean (SD) IMU SDI-Step	0.58 (0.12)	0.66 (0.09)	0.66 (0.05)	0.73 (0.27)	0.59 (0.08)	0.64 (0.05)
PB Slope (95% CI)	1.05 (1–1.17)	0.99 (0.94–1.06)	0.94 (0.74–1.12)	1 (1–1.16)	0.99 (0.97–1.01)	1 (0.91–1.09)
PB Intercept (95% CI)	−0.05 (−0.01–0.1)	0.03 (−0.03–0.05)	0.03 (−0.09–0.17)	0 (−0.09–0)	0.003 (−0.01–0.02)	0.005 (−0.05–0.06)
ICC (2,1) (95% CI)	0.97 (0.93–0.98)	0.9 (0.83–0.95)	0.84 (0.81–0.90)	0.98 (0.98–0.99)	0.94 (0.91–0.95)	0.94 (0.88–0.96)
BA–bias (95% LoA)	0.02 (−0.04–0.07)	0.02 (−0.06–0.09)	0.01 (−0.05–0.08)	0.01 (−0.05–0.06)	0 (−0.05–0.05)	0.01 (−0.04–0.06)
